# Colorectal cancer-related mutant *KRAS* alleles function as positive regulators of autophagy

**DOI:** 10.18632/oncotarget.5021

**Published:** 2015-09-25

**Authors:** Sara Alves, Lisandra Castro, Maria Sofia Fernandes, Rita Francisco, Paula Castro, Muriel Priault, Susana Rodrigues Chaves, Mary Pat Moyer, Carla Oliveira, Raquel Seruca, Manuela Côrte-Real, Maria João Sousa, Ana Preto

**Affiliations:** ^1^ CBMA - Centre of Molecular and Environmental Biology, Department of Biology, University of Minho, Campus de Gualtar, 4710-057 Braga, Portugal; ^2^ CNRS, UMR5095, University de Bordeaux 2, Bordeaux, France; ^3^ IPATIMUP - Institute of Molecular Pathology and Immunology of the University of Porto, Instituto de Investigação e Inovação em Saúde, Universidade do Porto, Porto, Portugal; ^4^ INCELL Corporation, San Antonio, Texas, USA

**Keywords:** autophagy, KRAS mutations, colorectal cancer, humanized yeast, non-cancer colon cells

## Abstract

The recent interest to modulate autophagy in cancer therapy has been hampered by the dual roles of this conserved catabolic process in cancer, highlighting the need for tailored approaches. Since RAS isoforms have been implicated in autophagy regulation and mutation of the *KRAS* oncogene is highly frequent in colorectal cancer (CRC), we questioned whether/how mutant *KRAS* alleles regulate autophagy in CRC and its implications. We established two original models, KRAS-humanized yeast and KRAS-non-cancer colon cells and showed that expression of mutated KRAS up-regulates starvation-induced autophagy in both. Accordingly, KRAS down-regulation inhibited autophagy in CRC-derived cells harboring *KRAS* mutations. We further show that KRAS-induced autophagy proceeds via up-regulation of the MEK/ERK pathway in both colon models and that KRAS and autophagy contribute to CRC cell survival during starvation. Since KRAS inhibitors have proven difficult to develop, our results suggest using autophagy inhibitors as a combined/alternative therapeutic approach in CRCs with mutant *KRAS*.

## INTRODUCTION

Colorectal cancer (CRC) is a leading cause of cancer-related death worldwide [1]. Recent therapies to treat metastatic CRC (mCRC) use EGFR pathway inhibitors in order to block tumor growth and proliferation [2, 3]. However, resistance still occurs, highlighting the need to develop alternative therapeutic approaches. In particular, mutations in *KRAS*, an oncogene with an important role in colon carcinogenesis, reportedly occur in 30% to 50% of CRCs and are frequently associated with resistance of mCRC to anti-EGFR therapies. Therefore, these therapies are only efficient in mCRC patients with wild type *KRAS* [4–8]. Autophagy has also emerged as a potential target for cancer therapy, as it has been implicated in the resistance of tumors to chemotherapy [9]. Autophagy is an evolutionary conserved catabolic process in eukaryotic cells from yeast to mammals that targets cellular components for lysosomal degradation to maintain cellular homeostasis [10]. Notably, cell lines harboring *RAS* mutations, such as those derived from bladder (T24, *HRAS^G12V^* mutation), lung (H1299, *NRAS^G12C^* mutation; H460, *KRAS^Q61H^* mutation), pancreatic (PANC-1, *KRAS^G12V^* mutation), and colorectal cancer (HCT116, *KRAS^G13D^* mutation), have high basal levels of autophagy [11]. Indeed, several reports implicated RAS isoforms in autophagy regulation, with different outcomes in cancer progression depending on the cell type [11–17]. However, little is known about the role of *KRAS* mutations in autophagy regulation in CRC cells and the signaling pathways involved.

In this work, we aimed to understand the precise role of mutant *KRAS* alleles in autophagy. For this purpose, we established two original cell models for a controlled genetic background where the only difference is the KRAS variant they express: non-cancer colon cells derived from normal human colon mucosal epithelium (NCM460) and a “humanized” *Saccharomyces cerevisiae*. The NCM460 cell line background does not contain *KRAS* mutations or deregulated pathways [18]. The yeast *S. cerevisiae*, a unicellular eukaryote highly amenable to manipulation and genetic modifications, is a well-established tool to study molecular aspects of different conserved biological processes, and a particularly advantageous system for heterologous expression [19, 20]. Moreover, autophagy-related (*ATG*) genes, responsible for the formation of the autophagosome, were first discovered in yeast and orthologues of many of these genes have been identified and characterized in higher eukaryotes, indicating that autophagy is a highly conserved pathway through evolution [21]. To transpose our findings to more clinically relevant cancer models, we used the reverse approach by silencing *KRAS* in two CRC-derived cell lines harboring different *KRAS* activating mutations. Here, we find that expression of mutant KRAS consistently increases autophagy in all models, which, in the colon cell lines, is associated with up-regulation of the MEK/ERK pathway. We also demonstrate that KRAS and autophagy support the survival of CRC cells exposed to stressful conditions like nutrient limitation. This suggests that inhibition of KRAS or autophagy may sensitize CRC cells harboring *KRAS* mutations to cancer therapies, reinforcing KRAS-induced autophagy inhibition as an emerging target for CRC therapeutic approaches.

## RESULTS

### *KRAS* mutations up-regulate autophagy during starvation in non-cancer colon cells and in a “KRAS-humanized yeast” model

To elucidate the role of oncogenic *KRAS* in autophagy regulation, we expressed FLAG-tagged wild-type KRAS (KRAS^WT^) or mutated KRAS (KRAS^G13D^, KRAS^G12D^ and KRAS^G12V^) in two model systems: in the human non-cancer colon NCM460 cell line, after stable infection, and in the yeast *S. cerevisiae ras2*Δ strain from the pCM184 plasmid, under the control of a *tet-off* promoter (this strain was chosen to abolish the input of yeast Ras2p, itself involved in autophagy regulation).

**Figure 1:**
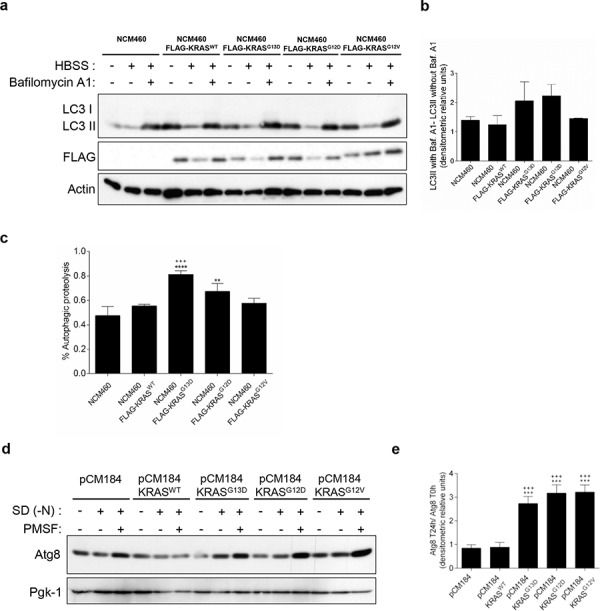

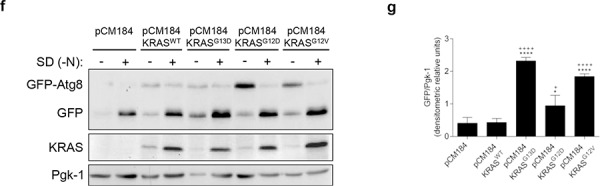
Activating *KRAS* mutations increase the autophagic flux during starvation, in NCM460 cells and *S. cerevisiae* **a.** Immunoblot analysis of LC3-I/II in NCM460 cells and NCM460 cells expressing *FLAG-KRAS^WT^*, *FLAG-KRAS^G13D^*, *FLAG-KRAS^G12D^* or *FLAG-KRAS^G12V^*. LC3-I expression levels were too low to be detected. FLAG immunoblot was used as a control for expression of FLAG-tagged KRAS constructs. **b.** LC3II/Actin ratios were determined and used to calculate the autophagic flux by subtracting the normalized LC3-II levels in the absence of Baf. A1 from the corresponding levels obtained in the presence of Baf. A1, according to guidelines already published. [24] Results are means ± SEM of three independent experiments. **c.** Level of autophagic proteolysis during starvation in NCM460 cell lines. Cells were incubated with L-[^14^C]valine, and chased for 6 h in complete medium, HBSS or HBSS supplemented with 0.1 μM Baf. A1. The percentage of autophagic proteolysis was determined by subtracting the levels of proteolysis of cells incubated in HBSS in the absence of Baf. A1 from the corresponding levels of proteolysis of cells incubated in HBSS supplemented with Baf. A1. Values represent mean ± SEM of three independent experiments. ^***^P < 0.001 vs NCM460; ^***^P < 0.001, *P < 0.05 vs NCM460 FLAG-KRAS^WT^; One-way ANOVA. **d.** Detection of Atg8p in the presence and absence of 1 mM PMSF in *S. cerevisiae* ras2Δ cells expressing the empty vector pCM184, pCM184/KRASWT, pCM184/KRASG13D, pCM184/KRASG12D or pCM184/KRASG12V after 24h of nitrogen starvation (SD -N) and **e.** Atg8 T24 h -N/Atg8 T0 h -N ratio after normalization of Atg8 to Pgk-1. **f.** Free GFP generated from the GFP-Atg8p fusion protein in *S. cerevisiae ras2*Δ cells co-expressing GFP-Atg8p with either empty vector pCM184, pCM184/KRAS^WT^, pCM184/KRAS^G13D^, pCM184/KRAS^G12D^ or pCM184/KRAS^G12V^ after 24 h of nitrogen starvation, as shown by immunoblot analysis. KRAS immunoblot was used as a control for expression of human KRAS. Pgk-1 immunoblot was used as a loading control. **g.** Free GFP/Pgk-1 ratio at 24 h of nitrogen starvation, using ImageJ software. Values are means ± SEM of three independent experiments. **P < 0.01; *P < 0.05 one-way ANOVA followed by Tukey's test vs pCM184/KRAS^WT^.

We first assessed the autophagic flux in NCM460 cells, by monitoring the conversion of LC3-I into LC3-II, a hallmark of autophagy, in the presence and absence of Bafilomycin A1 (Baf. A1) [22]. Overexpression of FLAG-KRAS^WT^, -KRAS^G13D^, -KRAS^G12D^ and -KRAS^G12V^ increased the basal level of LC3-II in complete medium, in comparison with parental NCM460 (non infected) cells (Figure 1a). During starvation, overexpression of FLAG-KRAS^G13D^ and -KRAS^G12D^, but not of -KRAS^G12V^, increased the autophagic flux in comparison with parental NCM460 and -KRAS^WT^- expressing cells, as shown by the increased accumulation of LC3-II upon inhibition of lysosomal degradation by Baf. A1 (Figures 1a, 1b and Supplementary Figure S1a). Though these differences were consistent, variability was high and did not reach statistical significance. We therefore quantitatively measured autophagic proteolysis of long-lived proteins radiolabeled with L-[^14^C]valine [23, 24]. In accordance with the previous assay, overexpression of FLAG-KRAS^G13D^ and -KRAS^G12D^, but not -KRAS^G12V^, increased the level of autophagic proteolysis, in comparison with parental NCM460 and -KRAS^WT^-expressing cells (Figures 1c and Supplementary Figure S1b). This increase was statistically significant in -KRAS^G13D^-expressing cells, but in -KRAS^G12D^- only when comparing with cells expressing -KRAS^WT^ (Figure 1c). Our results therefore indicate that some forms of mutated KRAS up-regulate autophagy in non-cancer colon cells.

In the *S. cerevisiae* model, autophagy levels were monitored by assessing the levels of the autophagic marker Atg8p, the yeast homologue of LC3, in the presence of the protease inhibitor PMSF to avoid degradation during the subsequent steps of the autophagy process [25]. No significant differences were observed in Atg8p levels between *ras2*Δ cells carrying the empty vector and *ras2*Δ cells expressing KRAS^WT^, after 24 h of nitrogen starvation (Figures 1d and 1e). In contrast, expression of KRAS^G13D^, KRAS^G12D^ and KRAS^G12V^ increased Atg8p levels under the same conditions (Figures 1d and 1e). These results show that activating *KRAS* mutations increase Atg8p levels more than wild-type *KRAS* in response to nitrogen starvation.

To confirm that the higher Atg8p levels detected were associated with induction of the autophagy flux, we also monitored the amount of Atg8p delivered to the vacuole. We transformed *ras2*Δ strains containing pCM184/KRAS plasmids or empty vector control with a GFP-*ATG8* construct and monitored the degradation of GFP-Atg8 to free GFP by vacuolar hydrolases, which reflects the level of autophagy, by immunobloting [26]. We found no significant differences in the accumulation of free GFP between *ras2*Δ cells carrying the empty vector and *ras2*Δ cells expressing KRAS^WT^, after 24 h of nitrogen starvation (Figures 1f and 1g). On the other hand, expression of KRAS^G13D^, KRAS^G12D^ and KRAS^G12V^ increased the levels of the free GFP moiety after 24 h of nitrogen starvation (Figures 1f and 1g). KRAS protein levels were confirmed by immunoblot against this specific human RAS isoform (Figure 1f). These results indicate that mutated KRAS lead to a higher delivery of Atg8p into the yeast vacuole during starvation than wild-type KRAS.

Altogether, our results in both non-cancer colon and “KRAS-humanized yeast” models show that mutated KRAS up-regulate autophagy to a higher extent than wild-type KRAS in response to nitrogen starvation, and validate both cell models to study the role of human KRAS proteins in the evolutionary conserved autophagic process.

### Suppression of *KRAS* by RNA interference inhibits autophagy in HCT116 and SW480 cell lines

**Figure 2:**
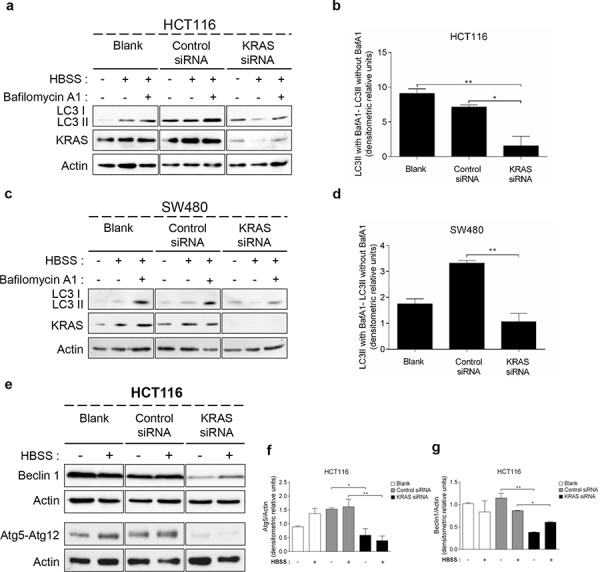

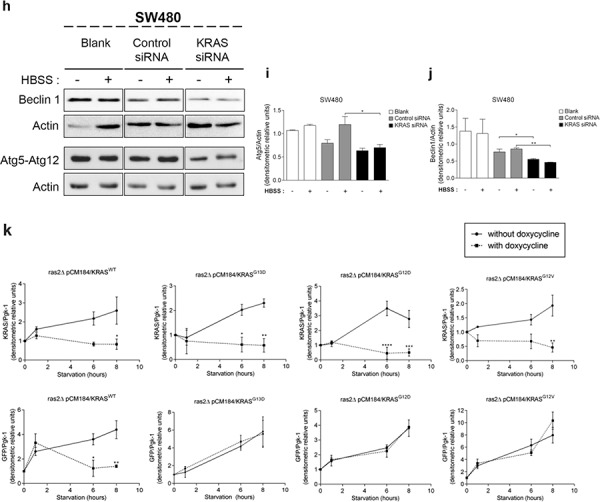
Suppression of *KRAS* in CRC- derived cell lines decreases autophagy **a. c.** Immunoblot analysis of LC3-I/II, **e.** Atg5-Atg12 conjugate (using anti-Atg5 antibody) and Beclin 1 in HCT116 and SW480 cell lines. Cells were left non transfected (blank) or transfected with either siRNA control or siRNA targeted against *KRAS*. After 48 h of transfection, the cells were maintained in complete medium or incubated in HBSS medium (starvation) for further 6 h in presence and absence of 20 nM Baf A1. LC3-I expression levels were too small to be detected. KRAS immunoblot was used to show the efficient silencing by *KRAS* siRNA. Actin immunoblot was used as a loading control. All the protein bands showed in the lanes are from the same immunoblot despite being organized in different boxes. [24] **b. d.** LC3-II/Actin ratios determined using *ImageJ* software were used to determine the autophagic flux ratio of by subtracting the normalized LC3-II levels in the absence of Baf. A1 from the corresponding levels obtained in the presence of Baf. A1, according to guidelines already published. [24] **f.** Atg5-Atg12/Actin ratio and **g.** Beclin 1/Actin ratio of HCT116 cells were determined using *Image J* software. Results are means ± SEM of three independent experiments. **P < 0.01; *P < 0.05 one-way ANOVA followed by Tukey's test. **h.** Atg5-Atg12 conjugate (using anti-Atg5 antibody) and Beclin 1 in SW480 cell lines. Cells were left non transfected (blank) or transfected with either siRNA control or siRNA targeted against *KRAS*. After 48 h of transfection, the cells were maintained in complete medium or incubated in HBSS medium (starvation) for further 6 h in presence and absence of 20 nM Baf A1. LC3-I expression levels were too small to be detected. KRAS immunoblot was used to show the efficient silencing by *KRAS* siRNA. Actin immunoblot was used as a loading control. All the protein bands showed in the lanes are from the same immunoblot despite being organized in different boxes. **i.** Atg5-Atg12/Actin ratio and **j.** Beclin 1/Actin ratio of SW480 cells were determined using *Image J* software. Results are means ± SEM of three independent experiments. **P < 0.01; *P < 0.05 one-way ANOVA followed by Tukey's test. **k.** Autophagy induction depends on the activating status of mutated KRAS activation during starvation in S. cerevisiae. KRAS/Pgk-1 and free GFP/Pgk-1 ratios of ras2Δ cells with/without expression of pCM184/KRAS^WT^, pCM184/KRAS^G13D^, pCM184/KRAS^G12D^ or pCM184/KRAS^G12V^. Cells were cultured in SC medium without the appropriate aminoacids + 2% glucose until exponential phase. At time 0, the cultures were subdivided: one part was incubated without doxycycline to maintain KRAS transcription active and another incubated with 10 μg/ml doxycycline to repress KRAS transcription. The level of free GFP generated and KRAS expression were assessed by immunoblot, at the indicated times and normalized for Pgk-1 using ImageJ software. Results are mean ± SEM of three independent experiments. ^****^P < 0.0001, ^***^P < 0.001, **P < 0.01, *P < 0.05 compared to the respective time point of cells non-treated with doxycycline.

In order to confirm the aforementioned results obtained in yeast and NCM460 cells, we used cell lines derived from CRC patients and assessed whether suppression of *KRAS* affects autophagy levels in HCT116 (*KRAS^G13D^*) and SW480 (*KRAS^G12V^*) cells. Indeed, suppression of *KRAS* by targeted small interference RNA (siRNA) in both cell lines significantly impaired the autophagic flux, evidenced by the decreased accumulation of LC3-II upon inhibition of lysosomal degradation by Baf. A1, whereas transfection with control siRNA did not (Figures 2a, 2b, Supplementary Figure S2a, 2c, 2d and Supplementary Figure S2b).

We next determined how depletion of *KRAS* affects the levels of other components of the autophagic machinery: the Atg5-Atg12 conjugate, a critical component of autophagosome formation [27], and Beclin 1, a component of the class III PI3K complex (PI3KC3) that induces autophagosome formation [28]. Depletion of *KRAS* by siRNA in SW480 and HCT116 cells decreased the levels of Atg5-Atg12 conjugate and Beclin 1 in comparison with control siRNA- transfected cells, both in complete medium and starvation conditions (Figures 2e–2j). Taken together, these data show that HCT116 and SW480 cells rely on *KRAS^G13D^* and *KRAS^G12V^* mutations, respectively, to maintain autophagy.

We also evaluated how manipulating the levels of mutant *KRAS* alleles in *S. cerevisiae* affects autophagy. For this purpose, cells grown in the absence of doxycycline (expressing KRAS) were transferred to nitrogen starvation media without or with doxycycline (to maintain or repress KRAS transcription, respectively) and the conversion of GFP-Atg8p to free GFP was monitored. As seen in Figures 2k and Supplementary Figure S3a, activation of *KRAS* transcription during starvation increased the level of wild-type KRAS, associated with an increase in the amount of free GFP generated, hence of autophagy induction. In turn, repression of wild-type *KRAS* transcription stabilized the level of KRAS protein and consequently the level of free GFP generated (Figures 2k and Supplementary Figure S3a). The same was not observed in yeast cells expressing KRAS^G13D^, KRAS^G12D^ or KRAS^G12V^. In these cells, nitrogen starvation maintaining KRAS expression also increased the levels of the respective KRAS variant, associated with accumulation of free GFP (Figures 2k, Supplementary Figure S3b, S3c and S3d). However, during repression of *KRAS* transcription, the level of KRAS proteins stabilized but the level of free GFP still increased (Figures 2k, Supplementary Figure S3b, S3c and S3d). Therefore, yeast cells harboring wild-type *KRAS* depend on the amount of KRAS protein for autophagy induction, whereas those harboring activating *KRAS^G13D^*, *KRAS^G12D^* or *KRAS^G12V^* do not. These results are consistent with constitutive activation of KRAS signaling due to the inability of mutated KRAS to hydrolyze GTP and consequently to be “switched off” [29–32], thus reproducing in the yeast background the effect of the aforementioned mutations on KRAS signaling.

These data also further support that *KRAS* mutations confer cells increased capacity for autophagy induction and that efficiently countering this effect can be achieved only by decreasing mutant KRAS protein levels below a certain threshold, as we observed in the CRC cell lines.

### *KRAS* mutations induce autophagy through up-regulation of the ERK pathway

**Figure 3:**
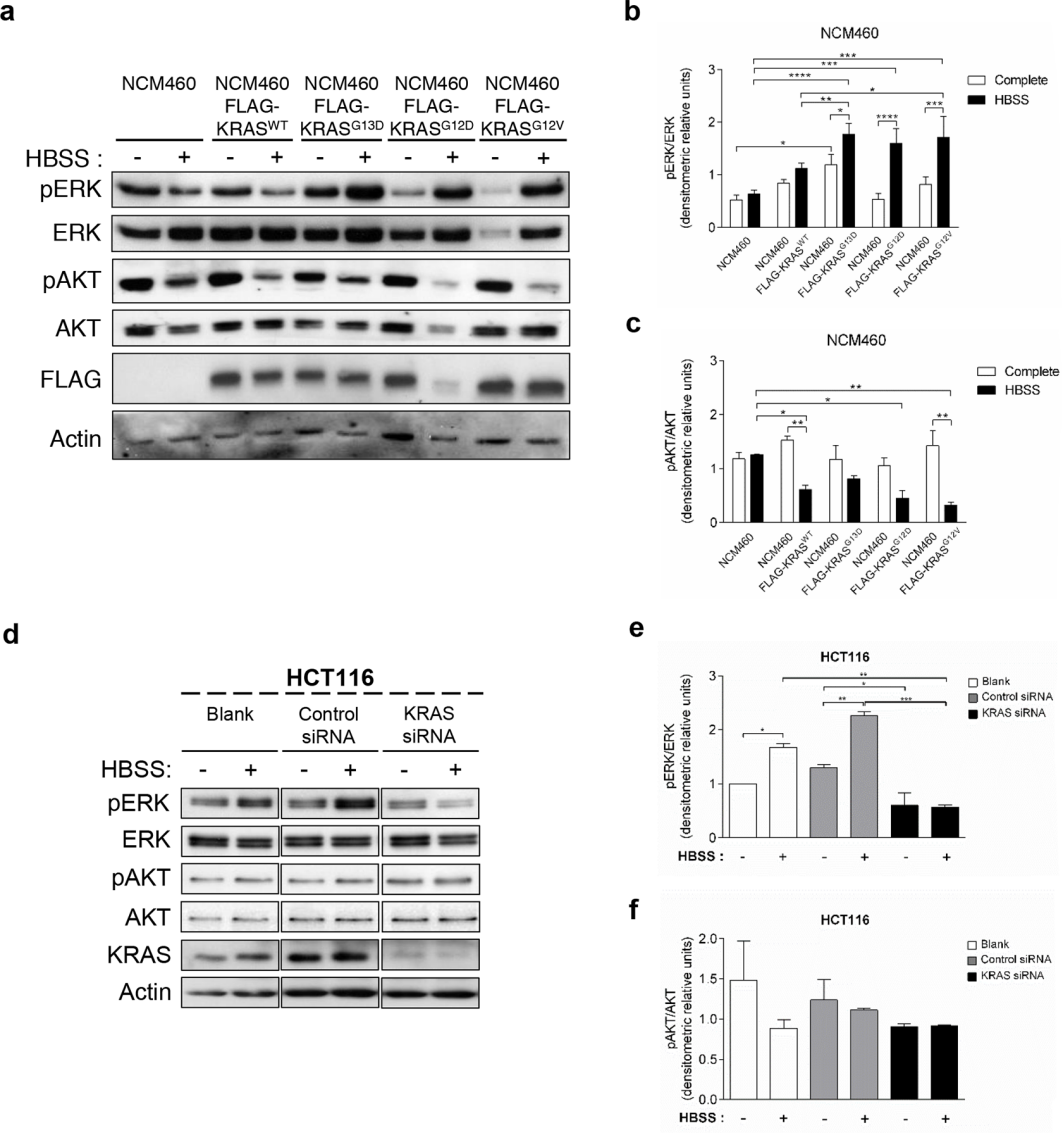

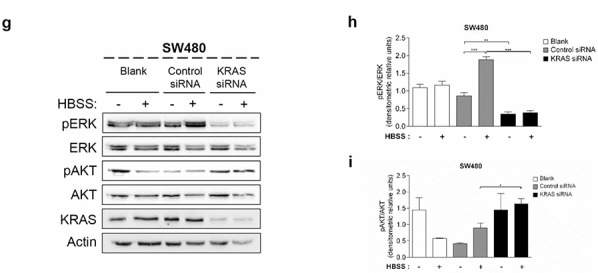
Mutated *KRAS* up-regulates ERK phosphorylation during starvation in normal and CRC- derived cells **a.** Immunoblot analysis of pERK and pAKT in parental NCM460 and NCM460 cells infected with *FLAG-KRAS^WT^*, *FLAG-KRAS^G13D^*, *FLAG-KRAS^G12D^* or *FLAG-KRAS^G12V^*. Cells were grown at a density of 80% and were maintained in complete conditions or incubated in HBSS (starvation) medium for 6 h. Cell lysates were analyzed by immunoblot for the indicated proteins. FLAG immunoblot was used as a control for expression of FLAG-tagged KRAS constructs. Actin immunoblot was used as a loading control. The **b.** pERK/ERK and **c.** pAKT/AKT ratios determined using *ImageJ* software. ^****^P < 0.0001; ^***^P < 0.001; **P < 0.01; *P < 0.05 one-way ANOVA followed by Tukey's test. **d.** Immunoblot analysis of pERK and pAKT in HCT116 cells. Cells were left non transfected (blank) or transfected with either siRNA control or siRNA targeted against KRAS. 48 h after transfection, the cells were maintained in complete medium or incubated in HBSS medium (starvation) for further 6 h and cell lysates were analyzed by immunoblot for the indicated proteins. KRAS immunoblot was used to show the efficient silencing by siRNA KRAS. Actin immunoblot was used as a loading control. All the protein bands showed in the lanes are from the same immunoblot despite being organized in different boxes. **e.** pERK/ERK and **f.** pAKT/AKT ratios were determined using *ImageJ* software. Results are means ± SEM of three independent experiments. ^***^P < 0.001; **P < 0.01; *P < 0.05 one-way ANOVA followed by Tukey's test. **g.** Immunoblot analysis of pERK and pAKT in SW480 cells. Cells were left non transfected (blank) or transfected with either siRNA control or siRNA targeted against *KRAS*. 48 h after transfection, the cells were maintained in complete medium or incubated in HBSS medium (starvation) for further 6 h and cell lysates were analyzed by immunoblot for the indicated proteins. KRAS immunoblot was used to show the efficient silencing by siRNA *KRAS*. Actin immunoblot was used as a loading control. All the protein bands showed in the lanes are from the same immunoblot despite being organized in different boxes. The **h.** pERK/ERK and **i.** pAKT/AKT ratios were determined using *ImageJ* software. Results are means ± SEM of three independent experiments. ^***^P < 0.001; **P < 0.01; *P < 0.05 one-way ANOVA followed by Tukey's test.

The MEK/ERK and the PI3K/AKT/mTOR pathways are the classical effectors of *RAS*, implicated in the regulation of various cellular responses known to generate resistance to CRC therapy [33]. We therefore analyzed how the *KRAS* variants affect the phosphorylation levels of ERK1/2 and AKT proteins in response to starvation. ERK phosphorylation increased in response to starvation in NCM460 cells overexpressing FLAG-KRAS^G13D^, -KRAS^G12D^ and -KRAS^G12V^, but not in parental NCM460 or FLAG-KRAS^WT^-expressing cells (Figures 3a and 3b). In contrast, starvation decreased AKT phosphorylation in NCM460 expressing all KRAS variants, but not in parental cells, though not to a significant extent in the case of FLAG-KRAS^G13D^ (Figures 3a and 3c).

In agreement with the results obtained in NCM460 cells overexpressing FLAG-KRAS^G13D^ and FLAG-KRAS^G12V^ (Figure 3a), starvation also increased ERK phosphorylation in both HCT116 (*KRAS ^G13D^* mutation) and SW480 (*KRAS ^G12V^* mutation) cells (Figures 3d, 3e, 3g and 3h). Accordingly, depletion of *KRAS* not only prevented this increase but also decreased ERK phosphorylation in non-starved cells (Figures 3d, 3e, 3g and 3h).

In HCT116 cells, AKT phosphorylation also mimicked the results obtained in the NCM460 FLAG-KRAS^G13D^ model: a slight but not significant decrease in control siRNA- transfected cells, and no difference in KRAS-depleted cells (Figures 3d and 3f). In SW480 control siRNA- transfected cells, no statistical significance differences were obtained in rich medium. On the other hand, under starvation conditions, depletion of *KRAS* increased AKT phosphorylation in comparison control siRNA- transfected cells also in agreement with the NCM460 FLAG-KRAS^G12V^ model (Figures 3g and 3i).

**Figure 4:**
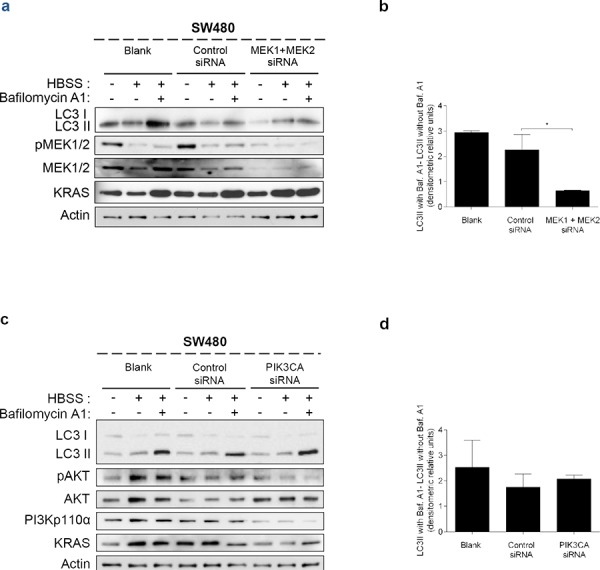
Suppression of MEK1+MEK2, but not of PIK3CA, decreases autophagy in SW480 cells Immunoblot analysis of LC3-I/II in SW480 cells, after suppression of **a.** *MEK1* and *MEK2* and **c.** *PIK3CA*. Cells were left non transfected (blank) or transfected with either control siRNA, siRNA targeted against *MEK1* and *MEK2* or siRNA targeted against *PIK3CA*. 48 h after transfection, the cells were maintained in complete medium or incubated in HBSS medium (starvation) for further 6 h and cell lysates were analyzed by immunoblot for the indicated proteins. MEK1/2 and PI3Kp110α immunoblot were used to show the efficient silencing by siRNA *MEK1/2* and siRNA *PIK3CA*, respectively. Actin immunoblot was used as a loading control. **b. d.** LC3-II/Actin ratios determined using *ImageJ* were used to calculate the autophagic flux by subtracting the normalized LC3-II levels in the absence of Baf A1 from the corresponding levels obtained in the presence of Baf A1. Results are means ± SEM of two independent experiments. *P < 0.05 one-way ANOVA followed by Tukey's test.

These data indicate that KRAS-induced autophagy is mediated through up-regulation of the MEK/ERK pathway and down-regulation of the PI3K/AKT pathway, known to activate the autophagy inhibitor mTOR [34], therefore resulting in autophagy induction. To confirm this hypothesis, we examined if these pathways affect starvation-induced accumulation of LC3-II after inhibition of lysosomal degradation by Baf. A1. In accordance with the aforementioned results, depletion of *MEK1/2* by siRNA decreased the autophagic flux of SW480 cells, whereas depletion of *PI3KCA* had no effect (Figures 4a, 4b, Supplementary Figure S4a, 4c, 4d and Supplementary Figure S4b).

Taken together, our results indicate that oncogenic KRAS proteins activate autophagy through up-regulation of the MEK/ERK pathway in non-cancer colon and CRC-derived cells, suggesting that autophagy plays an important role in KRAS-driven colorectal carcinogenesis.

### Suppression of *KRAS* or autophagy leads to cell death in CRC-derived cells

**Figure 5:**
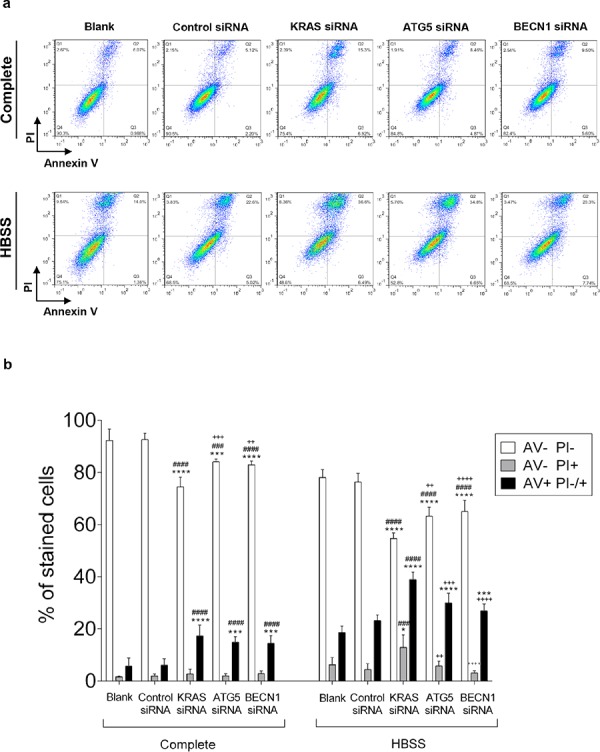
Suppression of *KRAS*, *ATG5* and *BECN1* promotes cell death in SW480 Cell death determined by Annexin V fluorescein isothiocyanate (AV-FITC) and propidium iodide (PI) assay in SW480 cells that were left non transfected (blank) or transfected with control siRNA, siRNA targeted against *KRAS*, *ATG5* or *BECN1*. 24 h after transfection, cells were maintained in complete medium or incubated in HBSS medium (starvation) for another 24 h. Cells were fixed and double stained with Annexin V and PI before analyzed by flow cytometry. **a.** Representative histograms of SW480 cells double-labeled with AV and PI. **b.** Quantitative analysis of AV/PI staining in SW480 cells. Percentages of apoptotic cells (AV+) are the sum of the lower and upper right panels (AV+ PI+/−). Values represent mean ±S.E.M. of at least three independent experiments. *P < 0.05; **P < 0.01, ^***^P < 0.001, ^****^P < 0.0001 comparing between each subset of cells (AV-PI-, AV-PI+; AV+ PI-/+). * vs Blank; # vs Control siRNA; + vs KRAS siRNA. Two-way ANOVA.

To understand whether KRAS and/or autophagy are required for CRC cell survival, prompting a possible therapeutic approach, we analyzed how depletion of *KRAS*, *ATG5* or *BECN1* by siRNA affected cell death. We found that depletion of *KRAS* in SW480 cells increased the number of cells stained with annexin V (AV+ PI −/+, early apoptosis plus late apoptosis/necrosis) in comparison with control siRNA- transfected cells, particularly evident under starvation conditions (Figure 5). Depletion of *ATG5* or *BECN1* (Supplementary Figure S5) had the same effect, though to a slightly lesser extent (Figure 5).

Taken together, our results show that KRAS-induced autophagy allows CRC-derived cells to survive in stressful conditions, such as nutrient limitation, as depletion of *KRAS* or impairment of the autophagic machinery promotes cell death of CRC cells.

## DISCUSSION

In the present study, we analyzed the role of activating *KRAS* mutations on autophagy regulation in CRC. We established a non-cancer colon NCM460 cell model to study, in the same genetic background, the effect of overexpressing three of the most common mutant *KRAS* alleles that occur in CRCs: KRAS^G13D^, KRAS^G12D^ and KRAS^G12V^ [35]. We show that autophagy is induced to a different extent depending on the mutant *KRAS* allele, since expression of KRAS^G13D^ and KRAS^G12D^ mutations up-regulated autophagy in NCM460 cells facing nutrient limitation, whereas wild-type KRAS or KRAS^G12V^ did not. In our “KRAS humanized yeast” system, KRAS^G13D^ and KRAS^G12D^ also induced autophagy in response to nitrogen starvation while wild-type KRAS did not. However, KRAS^G12V^ also highly up-regulated autophagy in this cell model. The lack of the genetic complexity found in human regulatory pathways and the lack of interference from wild-type KRAS and other human RAS family isoforms may explain the stronger phenotype observed in yeast compared with the human non-cancer colon cell model. Nonetheless, these results show that yeast is a particularly useful organism to address the function of KRAS in autophagy and that can be further explored to address other specific cellular functions of KRAS or KRAS localization within cellular compartments.

The results obtained in the yeast system show that expression of mutant KRAS *per se* can drive autophagy. However, the fact that expression of KRAS^G12V^ in NCM460 cells was not sufficient to up-regulate autophagy to the same extent as KRAS^G13D^ and KRAS^G12D^, suggests other factors may also play a role, depending on the cellular background. Accordingly, recent studies reported that induced expression of HRAS^G12V^ and KRAS^G12V^ can up-regulate autophagy in several cell models, such as mice kidney iBMK [11], breast MCF10A epithelial cells [15, 16], ovarian HOSE epithelial cells, MCF-7 cells and HUVECs cells [12]. However, it had not been addressed whether inhibition of this mechanism has an effect in tumor cells. We determined that depletion of *KRAS* by siRNA impairs the autophagic flux of CRC- derived cell lines HCT116 and SW480, harboring *KRAS^G13D^* and *KRAS^G12V^* mutations, respectively, indicating that autophagy of CRC cells harboring *KRAS* mutations depends on KRAS.

The best-characterized RAS effector pathways involved in autophagy regulation are the MEK/ERK and the PI3K/AKT/mTOR signaling pathways [33], which are commonly deregulated in CRC [36, 37]. However, it is not known if/how KRAS modulates these effectors to regulate autophagy in CRC. Here, we demonstrate that KRAS-induced autophagy is associated with up-regulation of ERK phosphorylation during nutrient limitation conditions in a non-cancer colon NCM460 cell model, consistent with a pro-autophagic role of MEK/ERK signaling [12, 38]. In accordance, depletion of *KRAS* by siRNA in the two CRC- derived cell lines used decreased activation of ERK. We further show that activation of the MEK/ERK pathway is critical for autophagy in SW480 cells, since inhibiting *MEK1/2* also significantly impaired this process. Overall, these results indicate that the MEK/ERK effector pathway is essential for KRAS-induced autophagy in CRC cells. However, activation of this pathway does not seem to be sufficient, since expression of KRAS^G12V^ in NCM460 cells induced phosphorylation of ERK, like KRAS^G12D^ and KRAS^G13D^, but did not up-regulate autophagy to a higher extent than wild-type KRAS, indicating KRAS may function through additional downstream effectors.

Overexpression of either KRAS variant in NCM460 cells had the opposite effect on phosphorylation of AKT during nutrient limitation conditions, which tended to decrease. However, there was no correlation with autophagy induction; indeed, this decrease was most significant in cells overexpressing KRAS^WT^ and KRAS^G12V^, which did not up-regulate autophagy. In agreement with the results obtained in the NCM460 model, depletion of *KRAS* in SW480 cells (*KRAS^G12V^* mutation) increased AKT activation during starvation. Since depletion of *KRAS* in this cell line decreased autophagy, this could suggest an anti-autophagic role of PI3K/AKT/mTOR signaling in CRC [13]. However, the results obtained in the HCT116 cell line, which displays constitutive activation of the AKT pathway due to an activating *PIK3CA^H1047R^* mutation, argue against this hypothesis. Indeed, on one hand autophagy is still induced in these cells; moreover, it is also decreased by depletion of *KRAS,* which is upstream of PI3K/AKT/mTOR. As expected, depletion of *KRAS* did not affect AKT activation in the HCT116 cell line, not only because these cells display constitutive activation of the AKT pathway, but also because overexpression of FLAG-KRAS^G13D^ in NCM460 cells did not affect pAKT levels. Overall, our results indicate the PI3K/AKT/mTOR pathway is not relevant in KRAS-induced autophagy. Accordingly, we found that depletion of *PIK3CA* did not affect autophagy induction of SW480 cells in response to starvation.

The interplay between RAS and autophagy on the survival of cancer cells is complex, as it may have both pro- and anti-tumorigenic roles. Previous studies demonstrated that expression of HRAS^G12V^ in MEFs and HRAS^G12V^ or KRAS^G12V^ in iBMK cells promotes cell proliferation through up-regulation of autophagy [11, 16, 39]. Others showed that HRAS^G12V^-induced autophagy contributes to cell death in HOSE, MCF-7 and HUVEC cells [12]. In our study, we sought to understand the effect of *KRAS*/autophagy inhibition on the cell death of CRC cells, which would attest to the clinical relevance of this process. We showed that depletion of *KRAS*, *ATG5* or *BECN1* results in cell death during growth of SW480 cells in complete medium, but to a higher extent in starvation conditions. These results indicate that autophagy mediated by activating *KRAS* mutations has a pro-survival role in CRC cells under stressful conditions, like nutrient deprivation.

EGFR inhibitors have been widely used in anti-cancer therapy; however, they are inefficient in mCRCs harboring activating *KRAS* mutations, which remains an obstacle to the effective treatment of mCRC [4–8]. Despite continuous efforts, no successful therapy targeting KRAS has been developed and it remains an elusive target for cancer therapy. This is due to the lack of specificity of KRAS inhibitors and the fact that KRAS can overcome inhibition of these targeted therapies [40, 41]. Direct inhibition of *KRAS* using small interference RNA (siRNA) may be an alternative approach to the use of chemical inhibitors and our results support this hypothesis. However, *in vivo* delivery issues of siRNA have yet to be overcome for widespread clinical applications, although efforts are being made to develop siRNA therapeutic approaches using lipid nanoparticle delivery systems [42, 43]. An alternative approach is to target downstream KRAS signaling. Autophagy is implicated as a potential mechanism of resistance to anticancer agents, as it aids in the response of tumor cells to cellular stress and/or increased metabolic demands related to increased cell proliferation [44]. Our results suggest that inhibition of autophagy can be an alternative or possibly combined therapeutic approach for the treatment of CRCs harboring activating *KRAS* mutations that depend on autophagy for survival. Indeed, studies in colon cancer xenografts using autophagy inhibitors in combination with chemotherapeutic agents showed that autophagy inhibition increases apoptosis accompanied by tumor regression [45]. Moreover, current clinical trials use the autophagy inhibitor hydroxychloroquine for treatment of a wide range of cancers, with promising results [46–50].

In summary, the results presented here contribute to a deeper understanding of the survival role of mutant *KRAS* alleles in CRC cells. We show that activating *KRAS* mutations increase autophagy in different cell models, and that this increase is mediated through activation of the MEK/ERK pathway in colon cells. We also demonstrate that inhibition of *KRAS* or autophagy can promote cell death in CRC cells harboring a *KRAS* mutation, highlighting KRAS-induced autophagy as an emerging target for CRC therapy.

## MATERIALS AND METHODS

### Cell lines and culture conditions

Cell lines used in this study were the noncancerous NCM460 cell line [18] and CRC-derived cell lines SW480 (*KRAS^G12V^* mutation) and HCT116 (*KRAS^G13D^* and *PIK3CA^H1047R^* mutations) [51] (ATCC). SW480 cells were grown in RPMI 1640 medium with glutamine (PAA, Austria) supplemented with 1% penicillin–streptomycin and 10% fetal bovine serum (FBS). HCT116 cells were grown in McCoy's 5A (PAA, Austria) supplemented with 2 mM L-glutamine, 1% penicillin–streptomycin and 10% FBS. NCM460 cells were grown in M3:10TM (INCELL, San Antonio, TX). All cell lines were cultured in a humidified incubator with 5% CO_2_ at 37°C. To assess basal protein levels, cells were cultured in complete medium and collected at 80% of confluence. For autophagy induction, cells growing in complete medium were washed three times with 1x PBS and incubated for 6 hours in Hank's Buffered Salt Solution (HBSS) buffered with 2.2 g/l NaHCO_3_. Bafilomycin A1 was added to inhibit lysosomal degradation in order to anaanalyze3-I/II protein levels (at 20 nM) or autophagic proteolysis (at 100 nM).

### Production of lentivirus containing *KRAS* genes and lentiviral infection

The human wild-type *KRAS* mRNA sequence was cloned into a pLenti6/V5 Directional TOPO^®^ (Invitrogen), giving rise to the *KRAS^WT^* expression vector. *KRAS^G13D^*, *KRAS^G12D^* or *KRAS^G12V^* mutant vectors were obtained by site-directed mutagenesis using specific primer sequences for each mutation. These vectors were used as templates to subclone wild-type and *KRAS* mutant sequences into the p3XFLAG-CMV vector to create FLAG-KRAS constructs before generating HIV-1 lentivirus-based vectors. A 5′ *Nhe*I site and a 3′*Pst*I site were introduced in the FLAG-KRAS sequence by PCR using p3XFLAG-CMV KRAS as a template and primers 5′-T TTGCTAGCATGGACTACAAAGACCATGACGG-3′ and 5′-TTTCTGCAGCTACATAATTACACACTTTGTC TT TGAC-3′. The PCR product was sub-cloned between the *Nhe*I and *Pst*I sites in pRRLSIN.cPPT.PGK.MSC.WPRE lentivector. Correct integration of FLAG-KRAS constructs was confirmed by sequencing. HEK-293FT cells were used as packaging cells, and virus production was as previously described [52]. NCM460 cells were infected with the lentiviral constructs pRR/FLAG-KRAS in complete medium, and cells collected after 5 days of infection. Infection with FLAG-KRAS was confirmed by immunofluorescence and by immunoblot of protein extracts, using an anti-FLAG primary antibody (Supplementary Figures S6a and S6b). All experiments using these cells were performed between the 4^th^ and 8^th^ weeks after infection. Population doubling determination showed a stable growth of cells with constitutive expression of FLAG-KRAS^WT^, -KRAS^G13D^, -KRAS^G12D^ or -KRAS^G12V^ (Supplementary Figure S6c).

### RNA silencing

HCT116 and SW480 cells were transiently transfected with siRNA using Lipofectamine 2000 (Invitrogen), according to manufacturer recommendations. siRNAs used were: *KRAS* siRNA (Hs_KRAS2_8), *ATG5* siRNA (Hs_APG5L_6) and *BECN1* siRNA (Hs_BECN1_5) designed by Qiagen; and *MAP2K1* siRNA (L-003571-00-0010), *MAP2K2* siRNA (L-003573-00-0010) and *PIK3CA* siRNA (L-003018-00-0010), designed by Dharmacon.

### Determination of long-lived protein degradation

Parental NCM460 cells and NCM460 cells infected with FLAG-KRAS constructs were labeled for 24 hours in complete medium containing 0.1 μCi L-[^14^C]valine/ml/well. After 24 hours, cells were washed three times with 1x PBS and radioactivity was pre-chased for 1 hour in complete medium in the presence of an excess of L-valine (10 mM) to remove the contribution of short-lived protein degradation. Cells were washed three times with 1x PBS, incubated for 6 hours either in complete medium or in HBSS in the presence or absence of Baf. A1 (0,1 μM) and with an excess of L-valine (10 mM). Supernatants were collected and free amino acids precipitated with 80% trichloroacetic acid (TCA), while proteins in adherent cells were precipitated with 10% TCA. The two fractions were transferred to vials containing 3 ml OptiPhase HiSafe II scintillation liquid (LKB Scintillation Products). Radioactivity was quantified in a liquid scintillation counter Packard Tri-Carb 2200 CA (Packard Instrument). Proteolysis was calculated as a percentage of the radioactivity in the supernatant relative to the total cell radioactivity.

### Annexin V/PI assay

SW480 cells were maintained in complete medium or incubated with HBSS medium. After 24 h of treatment, both suspended and attached cells were collected and washed in 1x PBS. 1 × 10^6^ cells were resuspended in 100 μl 1x binding buffer and incubated with 5 μl AV-fluorescein isothiocyanate (BD Biosciences, San Jose, CA, USA) and 10 μl 50 μg/ml PI for 15 min in the dark. Samples were analyzed in an Epics^®^ XL™ (Beckman Coulter) flow cytometer, equipped with an argon-ion laser emitting a 488-nm beam at 15 mW. Monoparametric detection of red fluorescence was performed using FL-4 (488/675 nm) and detection of green fluorescence was performed using FL-1 (488/525 nm). 20 000 cells were analyzed per sample and data analyzed using FlowJo software (version 7.6, Tree Star Inc., Ashland, OR, USA).

### Yeast plasmids

*KRAS^WT^*, *KRAS^G13D^*, *KRAS^G12D^* or *KRAS^G12V^* were sub-cloned as a *Bam*HI-*Apa*I fragments from pLenti6/V5-D-TOPO (Invitrogen) and inserted between the *Bam*HI and *Apa*I sites of pCM184 (*TRP1*) yeast plasmid, under the control of a *tet*-off promoter (repressed by the addition of doxycycline). The GFP-Atg8p (*URA3*) construct, for expression of *ATG8* under the control of its endogenous promoter in the pRS416 plasmid, was previously described [26].

### Yeast strains

The *Saccharomyces cerevisiae* mutant strain W303-1B *ras2*Δ *pho8*Δ*60* was generated by transformation of parental strain W303-1B *pho8*Δ*60* [53] with a disruption cassette amplified by PCR from genomic DNA of the BY4741 *ras2*Δ strain (Euroscarf). Correct integration of the cassette was confirmed by colony PCR. W303-1B *ras2*Δ *pho8*Δ*60* was co-transformed with pCM184/KRAS variants and pGFP-Atg8, and the resulting transformants grown in selective media lacking the appropriate amino acids. KRAS expression was confirmed by immunoblot against KRAS (Supplementary Figure S6d). No differences in growth of *S. cerevisiae ras2*Δ cells transformed either with empty vector or pCM184/KRAS variants were observed (Supplementary Figure S6e).

### Growth conditions

Yeast cells were grown aerobically in synthetic complete medium [SC; 0.17% yeast nitrogen base w/o amino acids and ammonium sulphate (Difco), 0.5% ammonium sulphate, 0.1% potassium phosphate, 0.2% Drop-out Mix and 0.01% auxotrophic requirements, pH 5.5] supplemented with 2% glucose in the presence of 10 μg ml^−1^ doxycycline. Cells were harvested, washed three times with water and resuspended in fresh medium without doxycycline to induce KRAS expression. For KRAS repression assays, 10 μg/ml doxycycline was added to the cultures to inhibit *KRAS* transcription. For nitrogen starvation assays, cells were grown in SC media without doxycycline until exponential phase, washed twice with distilled sterile water and suspended in nitrogen starvation media [SD-N; 0.17% yeast nitrogen base w/o amino acids and ammonium sulphate (Difco)] supplemented with 2% glucose for 24 h. All incubations were performed at 30°C, 200 r.p.m.

### Immunoblot analysis

Preparation of total protein extracts of human cell lines was performed as described in ref. [54]. Preparation of total protein extracts of yeast cells, SDS-PAGE and Western blots were performed as previously described [55].

### Antibodies

Antibodies used were: anti-actin, anti-Atg5, anti-LC3 and anti-FLAG (Sigma); anti-KRAS, and anti-yeast Atg8p (Santa-Cruz Biotechnology); anti-Beclin1, anti-phospho p44/42 MAPK (Thr202/Tyr204), anti-p44/42 total, anti-phospho Akt (Ser473), anti-Akt total, anti-PI3Kp110α, anti-phospho MEK1/2 and anti-MEK1/2 total (Cell Signalling); anti-yeast phosphoglycerate kinase (Pgk-1) (Molecular Probes); and mouse monoclonal GFP (1:3000; Roche Applied Science).

## SUPPLEMENTARY MATERIAL FIGURES


