# Supplementary material for: Colorectal cancer-related mutant KRAS alleles function as positive regulators of autophagy
Source: Oncotarget. 2015 Sep 25;6(31):30787–802. doi: 10.18632/oncotarget.5021 (PMC4741568; doi:10.18632/oncotarget.5021)
Supplement: Supplementary file 1 [file oncotarget-06-30787-s001.pdf]

SUPPLEMENTARY FIGURES

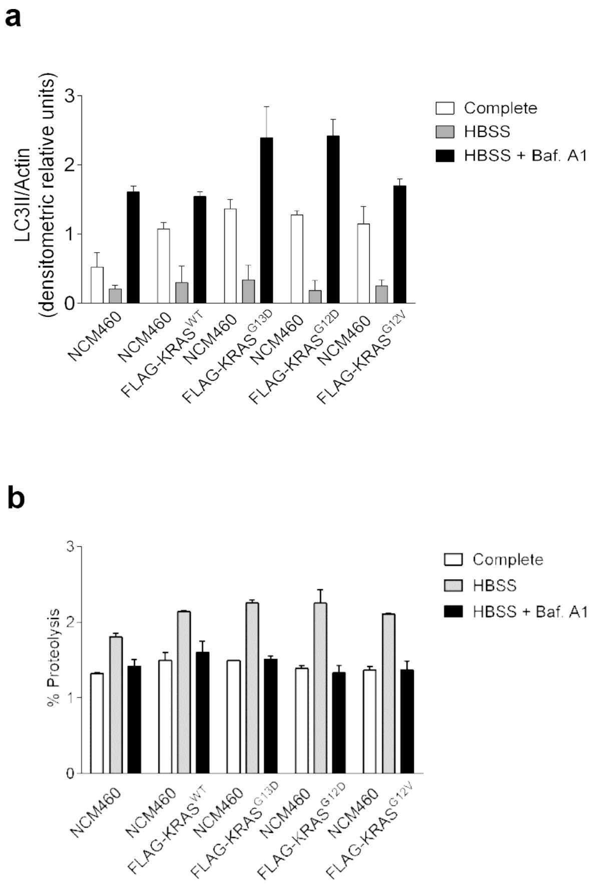

Supplementary Figure S1: Expression of mutated KRAS increases the autophagic flux during starvation in NCM460 cells. a. NCM460 cells and NCM460 cells expressing *FLAG-KRAS*^{WT}, *FLAG-KRAS*^{G13D}, *FLAG-KRAS*^{G12D} or *FLAG-KRAS*^{G12V} were subjected to immunoblot analysis of LC3-I/II. LC3-II/Actin ratio was determined using *ImageJ* software. Values are mean ± SEM of three independent experiments. **b.** Level of proteolysis in NCM460 cell lines. Cells were incubated with L-[^{14}C]valine, and chased for 6 h in complete medium (white bars), HBSS (gray bars) or HBSS supplemented with 0.1 µM Baf. A1 (dark gray bars). The data reports the stimulation of proteolysis relative to the respective basal levels measured under non-starvation conditions (white bars). Values are means of three independent experiments ± SEM.

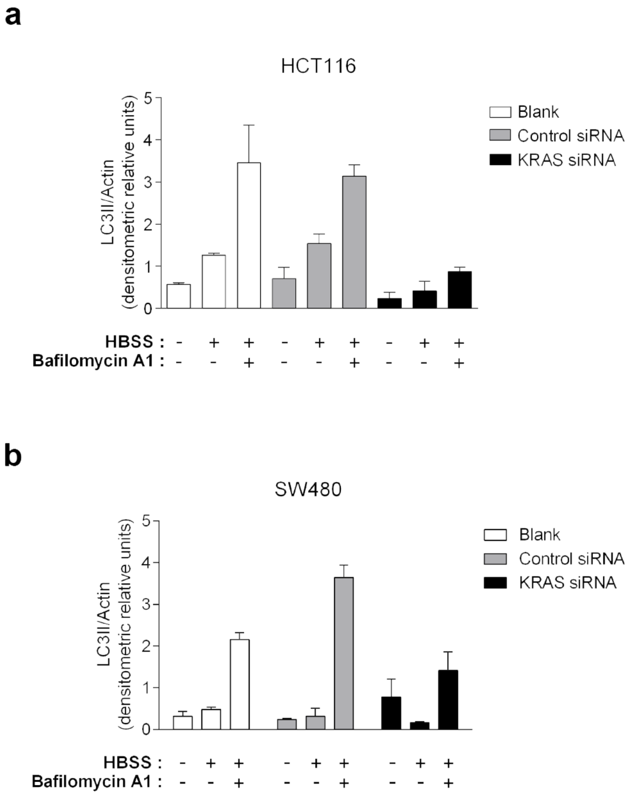

Supplementary Figure S2: Suppression of *KRAS* in CRC- derived cell lines decreases the level of autophagy. Detection of LC3-I/II by immunoblot analysis in *KRAS*-suppressed **a.** HCT116 and **b.** SW480 cells was used to determine LC3-II/Actin ratios using *ImageJ* software. Values are mean ± SEM of three independent experiments.

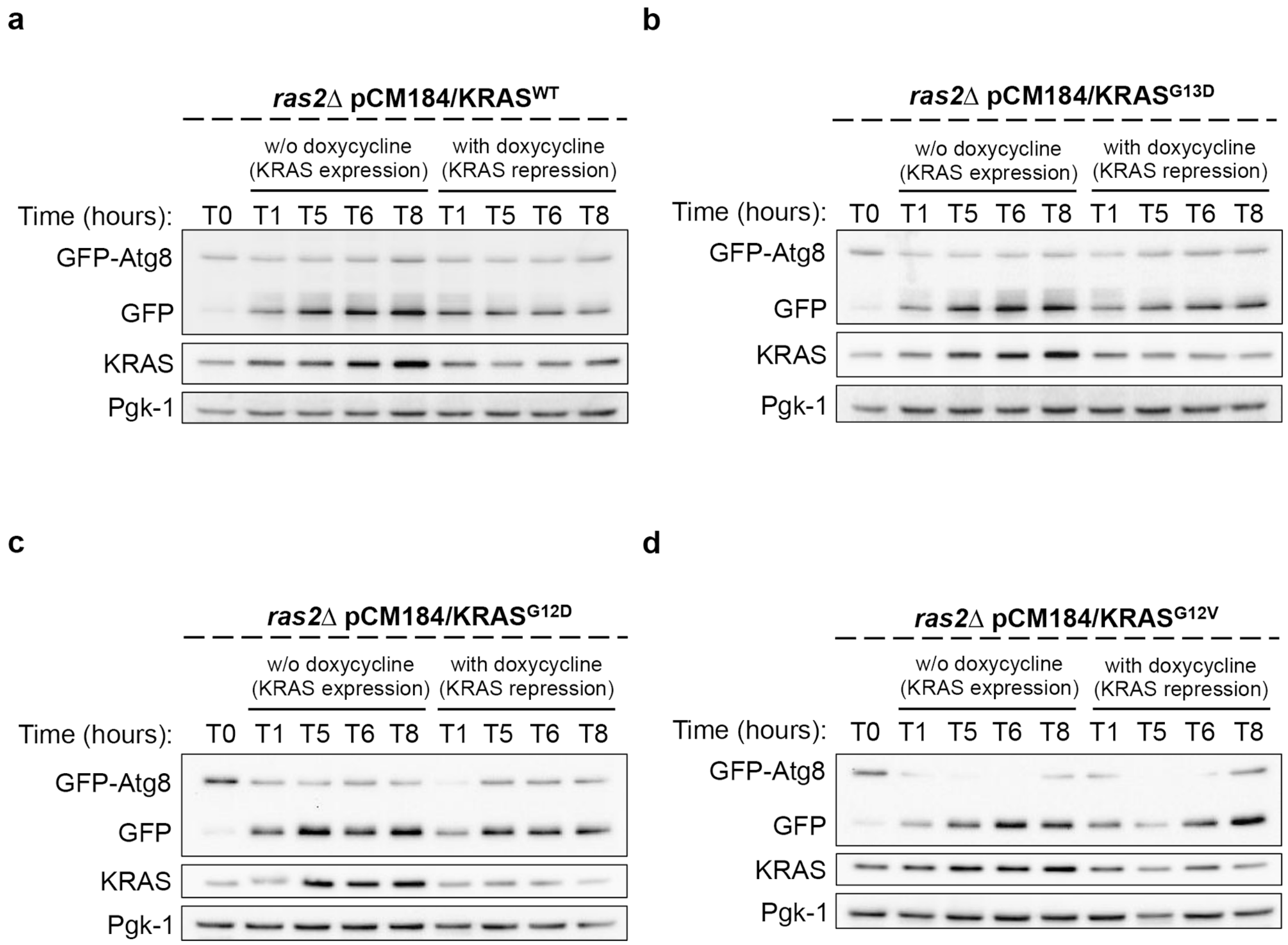

Supplementary Figure S3: Autophagy induction depends on activating status of mutated KRAS activation during starvation in *S. cerevisiae*. *ras2*Δ cells expressing or not the **a.** pCM184/KRAS^{WT}, **b.** pCM184/KRAS^{G13D}, **c.** pCM184/KRAS^{G12D} or **d.** pCM184/KRAS^{G12V}. The cells were cultured in SC medium without the appropriate aminoacids + 2% glucose until exponential phase. At time 0, the cultures were subdivided: one part of the culture was incubated without doxycycline to maintain KRAS transcription active and another incubated with 10 μg/ml doxycycline to repress KRAS transcription. Representative immunoblot of three independent experiments.

a

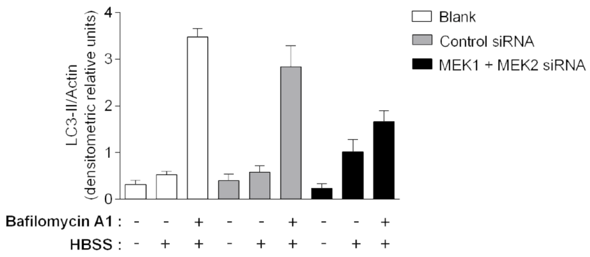

b

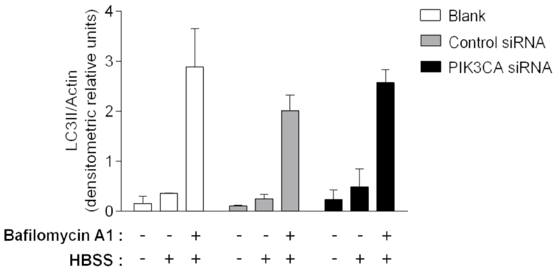

Supplementary Figure S4: Suppression of *MEK1* and *MEK2*, but not of *PIK3CA*, decreases autophagy in SW480 cells. Detection of LC3-I/II by immunoblot analysis in **a.** *MEK1/2*- and **b.** *PIK3CA*- suppressed SW480 cells was used to determine LC3-II/Actin ratios using *ImageJ*. Results are means ± SEM of two independent experiments.

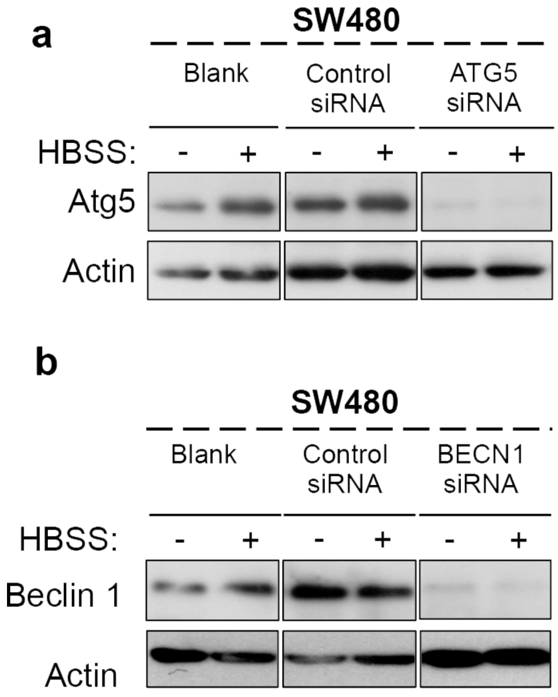

Supplementary Figure S5: Suppression of *ATG5* and *BECN1* in HCT116 and SW480 cell lines. Immunoblot analysis of **a.** Atg5-Atg12 conjugate (using anti-Atg5 antibody) and **b.** Beclin 1 in SW480 cells. Cells were left non transfected (blank) or transfected with control siRNA, siRNA targeted against *ATG5* or *BECN1*. 48 h after transfection, cell lysates were analyzed by immunoblot for the Atg5-Atg12 conjugate and Beclin 1. Actin as used as a loading control. All the protein bands showed in the lanes are from the same immunoblot despite being organized in different boxes.

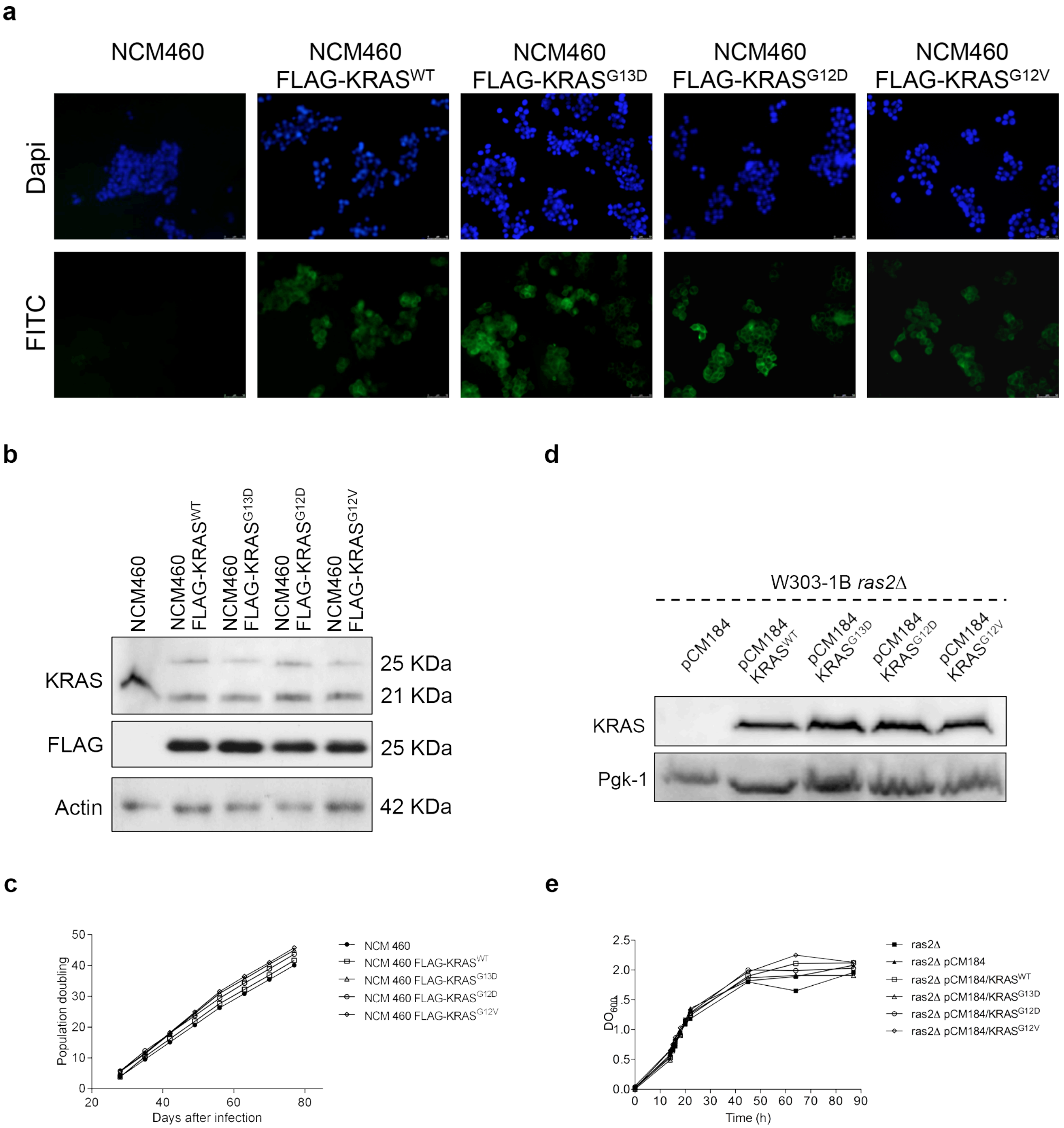

Supplementary Figure S6: Generation of non-cancer colon and humanized yeast cell models expressing mutated FLAG-KRAS. a. The NCM460 cell line was stably infected with *FLAG-KRAS*^{WT}, *FLAG-KRAS*^{G13D}, *FLAG-KRAS*^{G12D} or *FLAG-KRAS*^{G12V}. Infection of NCM460 cells with the KRAS constructs was confirmed by immunofluorescence against the FLAG tag, using fluorescent FITC secondary antibody. Infected cells are depicted as green-labeled cells whereas non-infected cells are not labeled. DAPI was used for nuclei staining. Observation with 40x objective. **b.** The protein levels of FLAG-tagged KRAS^{WT}, KRAS^{G13D}, KRAS^{G12D} and KRAS^{G12V} were analyzed by immunoblot against FLAG (for detection of a 25KDa product – exogenous KRAS) or KRAS (for detection of a 25 KDa product – exogenous KRAS; and a 21 KDa product – endogenous KRAS). Actin was used as a loading control. **c.** NCM460 cell lines stably infected with *FLAG-KRAS*^{WT}, *FLAG-KRAS*^{G13D}, *FLAG-KRAS*^{G12D} or *FLAG-KRAS*^{G12V}, were routinely plated at 1×10^6 cells/plate every week. After 7 days, the number of cells was scored and used to calculate the number of times the population doubled. **d.** Immunoblot analysis of KRAS in W303-1B *ras2Δ pho8Δ60* cells carrying the empty vector pCM184, pCM184/KRAS^{WT}, pCM184/KRAS^{G13D}, pCM184/KRAS^{G12D} or pCM184/KRAS^{G12V}. Cells were grown until exponential phase in the absence of doxycycline for induction of KRAS expression. Pgk-1 immunoblot was used as a loading control. **e.** *ras2Δ* and *ras2Δ* carrying pCM184, pCM184/KRAS^{WT}, pCM184/KRAS^{G13D}, pCM184/KRAS^{G12D}, pCM184/KRAS^{G12V} were cultured in SC medium + 2% glucose. Culture density was assessed by spectrophotometric measurement of the DO_{600} at the indicated times.
